# Muscle Oxygen Saturation Responses During Maximal and Submaximal Exercise According to *SLC16A1 (MCT1)* Gene Polymorphism in Long-Distance Runners: A Cross-Sectional Pilot Study

**DOI:** 10.3390/genes16111324

**Published:** 2025-11-03

**Authors:** Shotaro Seki, Tetsuro Kobayashi, Naoki Kikuchi, Kosaku Hoshina, Inkwan Hwang

**Affiliations:** 1 Faculty of Sport Science, Nippon Sport Science University, Tokyo 158-8508, Japan; n.kikuchi@nittai.ac.jp (N.K.); hwang@nittai.ac.jp (I.H.); 2 Department of Education, Ikuei University, Takasaki 370-0011, Gunma, Japan; 3 Graduate School of Media and Governance, Keio University, Fujisawa 252-0882, Kanagawa, Japan; kosaku@sfc.keio.ac.jp

**Keywords:** distance runner, aerobic capacity, blood lactate, genetics, polymorphism, recovery, physiological response, muscle oxygen

## Abstract

**Background:** Blood lactate concentration and muscle oxygen saturation (SmO_2_) are widely used indicators of endurance performance, reflecting the balance between oxygen delivery and utilization during exercise. To date, no studies have examined how the rs1049434 polymorphism of the *SLC16A1* gene (the polymorphism) influences SmO_2_ referenced to blood lactate thresholds in long-distance (LD) runners. This pilot study aimed to investigate the association between SmO_2_ referenced to blood lactate concentration during maximal and submaximal exercise and the polymorphism in male collegiate LD runners. **Methods:** Overall, 15 Japanese male collegiate LD runners participated. Physiological parameters, including respiratory gas data, were measured during a graded incremental exercise test using the breath-by-breath method. SmO_2_ was recorded from the right vastus lateralis muscle. Participants were genotyped for rs1049434, and comparisons were made between the AA genotype and T-allele carriers (AT + TT genotype). **Results:** Runners with the AA genotype exhibited significantly higher %$\overset{\cdot }{V}$O_2max_ at the lactate threshold (*p* = 0.044) and at the onset of blood lactate accumulation (OBLA) than T-allele carriers (*p* = 0.023). For SmO_2_ measurements, those with the AA genotype displayed shorter t1/2_reoxygenation_ (*p* = 0.043) and higher SmO_2max_ (*p* = 0.045). Furthermore, SmO_2_-OBLA was significantly higher in the AA genotype than in T-allele carriers (*p* = 0.029). **Conclusions:** These findings suggest that runners with the AA genotype may have greater oxygen utilization efficiency and potentially improved muscle oxygen delivery during high-intensity exercise. However, these are preliminary results, and further studies with larger and more diverse cohorts are needed to confirm these observations.

## 1. Introduction

Blood lactate concentration has been widely utilized to assess the training load and running performance in long-distance (LD) runners. Representative indices include running velocity at the lactate threshold (LT), velocity at the onset of blood lactate accumulation (OBLA) [1,2], and the maximal lactate steady state [3]. Furthermore, the capacity to metabolize lactate, which serves as a primary energy substrate during exercise, is a critical determinant of endurance performance.

Lactate serves as an energy substrate during exercise and at rest in various organs, including skeletal muscles, red blood cells, and the brain. Its metabolic turnover can even exceed that of glucose during moderate- to high-intensity exercise [4]. Lactate transport across cell membranes is primarily mediated by monocarboxylate transporters MCT1 and MCT4 [5,6,7]. These transporters facilitate bidirectional movement depending on the prevailing concentration gradient [8,9,10]. The above results suggest that enhanced functional activity of MCT1 may improve lactate transport capacity, thereby playing an important role in sustaining energy supply and performance in LD runners.

Since the early 2000s, studies have reported that the transport capacity of MCT1 is influenced by genetic variation. The T1470A polymorphism of the *SLC16A1 (MCT1)* gene (rs1049434 polymorphism), located on the short arm of chromosome 1p12, is a missense mutation that leads to the substitution of glutamic acid with aspartic acid at codon 490 [11]. Individuals with AA genotype exhibit approximately 60–65% greater lactate transport capacity than those carrying the AT or TT genotype [11]. Consistent findings have been reported in C2C12 myoblasts, where A-allele carriers (AA + AT genotypes) demonstrated higher lactate transport activity than T-allele carriers (AT + TT genotypes) [12]. These genotype-related differences in lactate transport have prompted research into their association with athletic status and performance across diverse sports, ranging from endurance to power events. Indeed, T-allele carriers are commonly associated with power and sprint athletes [13,14], whereas AA genotype and A-allele carriers are linked to endurance and intermittent athletes [15,16]. In intermittent athletes, AA genotype has been reported to contribute to faster post-exercise lactate clearance compared with TT genotype [17]. Furthermore, as a general indicator of aerobic capacity, maximal oxygen uptake ($\overset{\cdot }{V}$O_2max_) is significantly higher in the AA genotype than in the TT genotype among endurance athletes [18]. Collectively, these findings highlight the potential of AA genotype that may enhance metabolic efficiency and exercise tolerance in various forms of endurance exercise. In LD runners, lactate transport is a critical determinant of their performance. Previous studies have reported a significantly higher prevalence of AA genotype in elite runners than in controls or sub-elite runners [19,20]. Moreover, physiological performance indices such as $\overset{\cdot }{V}$O_2max_ are greater in AA genotype than in T-allele carriers [20]. Collectively, these findings suggest that the expression of AA genotype may contribute to a more efficient energy supply and confer favorable physiological performance characteristics in LD runners.

In recent years, near-infrared spectroscopy (NIRS) has been widely adopted as a noninvasive tool to assess skeletal muscle oxygenation, with muscle oxygen saturation (SmO_2_) as a key metric. SmO_2_ reflects the balance between oxygen delivery and consumption at the microvascular level, integrating both hemoglobin oxygen dissociation and myoglobin desaturation [21,22]. Previous studies have reported that SmO_2_ exhibits a strong association with blood lactate concentrations, a widely used marker in sports performance assessment [23], and demonstrates reliability comparable to that of oxygen uptake and heart rate [24]. Therefore, NIRS is expected to serve as a practical alternative to more invasive methods in competitive sports settings. Furthermore, studies in the general population have suggested that SmO_2_ may differ according to rs1049434 polymorphism, which influences lactate transport capacity [25]. However, to date, no studies have investigated the relationship between SmO_2_ referenced to blood lactate concentrations and rs1049434 polymorphism in LD runners. Therefore, this pilot study aimed to investigate the association between SmO_2_ referenced to blood lactate concentration as measured during maximal and submaximal exercise and the rs1049434 polymorphism in male collegiate LD runners. We hypothesized that the AA genotype would exhibit higher SmO_2_ during submaximal exercise and faster post-exercise recovery than the T-allele carriers.

## 2. Materials and Methods

### 2.1. Study Design

This study was a cross-sectional investigation of male collegiate LD runners. Data were collected in March 2025. Physiological responses during maximal and submaximal exercise were assessed using the blood lactate curve and $\overset{\cdot }{V}$O_2max_ tests. Simultaneously, SmO_2_ in the right vastus lateralis was recorded using NIRS throughout both testing protocols. Saliva samples for genotyping were collected after the completion of physiological tests, following a period of rest until the heart rates of participants returned to baseline. Basic demographic and training-related information was acquired via a questionnaire distributed at the same time. Genotyping of the retrieved saliva samples was performed after the completion of all physiological measurements to avoid potential sampling bias.

### 2.2. Participants

**Figure 1:**
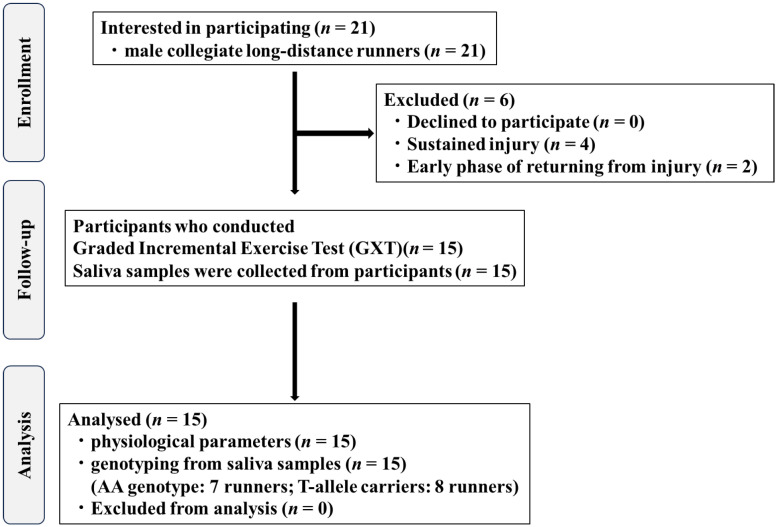
Flow diagram showing the inclusion and exclusion of study participants.

This study included 15 male collegiate LD runners (age: 20.6 ± 0.9 years; training experience: 6.9 ± 1.9 years; subcutaneous fat thickness of the vastus lateralis: 6.4 ± 1.2 mm). The physical characteristics of the participants and their 5000-m personal best records (PBRs), stratified by genotype, are presented in Table 1. The flow of participants is illustrated in Figure 1.

This study was conducted in accordance with the principles of the Declaration of Helsinki. All participants were fully informed about the purpose, procedures, and potential risks of the study before participation, and written informed consent was obtained. The study protocol was approved by the Ethics Committee for Human Research of the Nippon Sports Science University (approval no.: 023-H120; approval date: 18 October 2023).

### 2.3. Experimental Procedures

#### 2.3.1. Questionnaire

Information on participants’ age, years of training experience, competition level, and personal best record for the 5000-m was collected using a structured questionnaire.

#### 2.3.2. Genotyping

Saliva samples (2 mL) were collected using a self-collection kit (Oragene DISCOVER; DNA GenoTek, Stittsville, ON, Canada). Samples were incubated in a water bath at 55 °C for 60 min (SN-100SD, NISSIN, Tokyo, Japan), and DNA was extracted following the manufacturer’s instructions. After incubation, 500 μL of each saliva sample was transferred to a microcentrifuge tube, and 20 μL (1/25th of the saliva volume) of Oragene DNA Purifier (PT-L2P, DNA Genotek) was added. The samples were incubated on ice for 10 min and centrifuged at 15,000× *g* for 5 min at room temperature (15–30 °C). The supernatant was transferred to a new tube, mixed with an equal volume of 100% ethanol, and the DNA was precipitated for 10 min, followed by centrifugation at 15,000× *g* for 2 min. The supernatant was discarded, and the pellet was washed and resuspended in 100 μL of TE buffer. Genotyping of the rs1049434 polymorphism was performed using TaqMan™ SNP assays (Assay ID: C___2017662_30) on a CFX96 Touch™ Real-Time polymerase chain reaction (PCR) system (Bio-Rad, Hercules, CA, USA). FAM and VIC fluorescence signals corresponded to the A and T alleles, respectively, with increased signal intensity indicating homozygous genotypes (AA or TT), whereas concurrent increases in both signals denoted a heterozygous genotype (AT). Genotypes were determined from endpoint fluorescence scatter plots and classified using CFX Manager software (version 2.1; Bio-Rad). PCRs were prepared using 2.5 μL of TaqMan™ Universal Master Mix II, 0.125 μL of TaqMan™ SNP Genotyping Assay mix, 2.375 μL of nuclease-free water, and 1 μL of genomic DNA.

#### 2.3.3. Body Composition Measurement

Body composition was assessed using a bioelectrical impedance analyzer (InBody 430; InBody, Seoul, Republic of Korea). Height was measured using a stadiometer (Silver Wide YS101-S; Yoshida Manufacturing, Osaka, Japan). The following parameters were recorded: body weight, fat mass, fat-free mass, body fat percentage, and body mass index.

#### 2.3.4. Graded Incremental Exercise Test (GXT)

The GXT was performed to assess blood lactate levels and $\overset{\cdot }{V}$O_2max_. A 4-min warm-up was conducted, starting at 200 m/min with speed rising by 20 m/min every minute. Participants wore a face mask (Hans Rudolph, Shawnee, KS, USA), and physiological variables were measured breath by breath method using a pulmonary exercise monitoring system (AE 310S, Minato Medical Science, Osaka, Japan). The ambient temperature and humidity were maintained at 20 °C and 50%, respectively. The gas analyzer was calibrated before each test using reference gases (ambient air: O_2_ 20.93%, CO_2_ 0.04%, N_2_ balance; expired gas equivalent: O_2_ 15.08%, CO_2_ 5.02%, N_2_ balance). Ventilation (VE) was quantified with a hot-wire flowmeter, which was calibrated using a 2-L flow calibrator (ACA105, Minato Medical Science, Osaka, Japan). The heart rate was simultaneously monitored using a heart rate sensor (H10, Polar Electro, Kempele, Finland). Following the synchronization of the heart rate sensor and pulmonary monitoring system, the participants rested for 1 min before the test began. The test commenced at 240 m/min. Each stage involved 3 min of steady running, followed by a 1-min rest, with the running speed incremented by 20 m/min per stage until blood lactate concentration exceeded 4 mmol/L. During the 1-min rest intervals, ratings of perceived exertion (RPE) and blood lactate concentration (Lactate Pro 2, Arkray, Kyoto, Japan) were recorded. When the blood lactate level exceeded 4 mmol/L, the participants rested for 2 min before commencing the $\overset{\cdot }{V}$O_2max_ test. The $\overset{\cdot }{V}$O_2max_ test was started from the stage immediately before the blood lactate concentration exceeded 4 mmol/L. Running speed was then increased by 10 m/min every 30 s until the participant could maintain the pace for approximately 60–90 s, after which the test proceeded to volitional exhaustion. Blood lactate concentrations were measured immediately after exercise and again at 3 and 5 min post-exercise.

$\overset{\cdot }{V}$O_2max_ was determined as the maximal oxygen uptake ($\overset{\cdot }{V}$O_2_) measured over 30-s intervals. At least three of the following criteria were used to verify $\overset{\cdot }{V}$O_2max_: (1) plateauing of $\overset{\cdot }{V}$O_2_, (2) heart rate attaining the estimated maximal heart rate (220–age ± 5 bpm), (3) respiratory exchange ratio ≥ 1.15, (4) RPE ≥ 18, and (5) post-exercise blood lactate concentration ≥ 10 mmol/L.

#### 2.3.5. SmO_2_ Measurement

Peripheral SmO_2_ was measured using a small NIRS sensor (MOXY, Fortiori Design LLC, Hutchinson, MN, USA) placed on the belly of the right vastus lateralis, following the attachment method described by Porter et al. [26] (Figure S1). The sensor was enclosed in a flexible polyurethane skirt to prevent interference from ambient light. Both the sensor and the skirt were secured to the muscle belly using a pre-cut adhesive tape (BSN Medical Ltd., Hull, UK).

The NIRS sensor emitted light at four wavelengths (680, 720, 760 and 800 nm) and measured the returning light using two detectors positioned 12.5 mm and 25 mm away from the light source. This setup enabled the evaluation of local muscle blood flow and oxygenation, based on changes in the total hemoglobin concentration in the capillaries as an index. Because the penetration depth is approximately half the distance between the light source and the detector [27], the effective penetration depth of the sensor is 12.5 mm. Therefore, the maximum allowable subcutaneous fat thickness was set to 12.5 mm, and the subcutaneous fat thickness in the right thigh was measured using calipers (Subcutaneous Fat Caliper; Meiko Co., Ltd., Tokyo, Japan). SmO_2_ data were collected at 0.5 Hz and stored in the internal memory of the sensor. Data smoothing was performed using a second-order zero-phase low-pass Butterworth filter with a cutoff frequency of 0.03 Hz. The placement procedure was standardized for all participants by the same examiner to ensure measurement consistency.

### 2.4. Calculated Variables

$\overset{\cdot }{V}$O_2_ at the LT ($\overset{\cdot }{V}$O_2_-LT) was determined as described by Beaver et al. [28]. The slopes of linear portions before and after the breakpoint in the blood lactate curve were calculated, and their intersection was defined as $\overset{\cdot }{V}$O_2_-LT. $\overset{\cdot }{V}$O_2_ at the onset of OBLA ($\overset{\cdot }{V}$O_2_-OBLA) was defined as oxygen uptake corresponding to a blood lactate concentration of 4 mmol/L. The percentages of $\overset{\cdot }{V}$O_2_-LT and $\overset{\cdot }{V}$O_2_-OBLA relative to $\overset{\cdot }{V}$O_2max_ (%$\overset{\cdot }{V}$O_2max_ at LT and %$\overset{\cdot }{V}$O_2max_ at OBLA, respectively) were calculated.

SmO_2_ at the LT (SmO_2_-LT) and at OBLA (SmO_2_-OBLA) were calculated using the same method as $\overset{\cdot }{V}$O_2_-LT and $\overset{\cdot }{V}$O_2_-OBLA. Resting SmO_2_ (SmO_2rest_) was defined as the mean value during the 3-min seated rest period before the blood lactate curve test. Maximum muscle oxygen saturation (SmO_2max_) was defined as the highest SmO_2_ recorded from the onset of reoxygenation after the $\overset{\cdot }{V}$O_2max_ test until test completion. Minimum muscle oxygen saturation (SmO_2min_) was defined as the lowest SmO_2_ detected immediately before reoxygenation. Reoxygenation time (t1/2_reoxygenation_) was calculated as the time required for SmO_2_ to increase from SmO_2min_ to 50% of the difference between SmO_2min_ and SmO_2max_ [29].

### 2.5. Statistical Analysis

Physiological variables are expressed as means ± standard deviation. The association between the 5000-m PBR and each physiological variable was analyzed using Pearson’s product-moment correlation coefficient. Normality was assessed using the Shapiro–Wilk test (Table S1). Parametric analyses using unpaired *t*-tests were conducted when the normality assumption was satisfied. Outcomes are reported with *p*-values and 95% confidence intervals (CIs), and effect sizes were quantified using Cohen’s *d*, categorized as small (0.2–0.6), medium (0.6–1.2), large (1.2–2.0), or very large (≥2.0) [30]. The 95% CIs of effect sizes were also reported. For non-normally distributed data, Mann–Whitney U-tests were used, with *p*-values, 95% CIs, and effect sizes calculated. Effect sizes were expressed as *r* and interpreted as small (0.1–0.3), medium (0.3–0.5), or large (≥0.5) [31].

This is the first study to investigate the association between SmO_2_ responses during maximal and submaximal exercise and lactate metabolism–related gene polymorphisms in male collegiate LD runners and was therefore considered a pilot study. A priori sample size estimation was performed using G*Power 3.1 (version 3.1; Heinrich Heine University Düsseldorf, Düsseldorf, Germany). For an independent two-group *t*-test with an effect size of *d* = 1.2, α = 0.05, and power = 0.80, a total of 24 participants (12 per group) were required. However, due to training camps and competitions throughout the year, and because athletes who were injured or in the immediate post-injury phase were excluded, recruiting the estimated number of the participants was challenging (Figure 1). Consequently, the study was conducted with 15 participants (AA genotype carriers: *n* = 7; T-allele carriers: *n* = 8). Post hoc statistical power for the independent *t*-test and the Mann–Whitney U-test was calculated using G*Power (Table S2). All statistical analyses were performed using SPSS (version 29.0; IBM Corp., Armonk, NY, USA), and the significance level was set at *p* < 0.05.

## 3. Results

### 3.1. Correlation Between the PBR and Physiological Parameters in the Participants

The correlation between the 5000-m PBR and physiological parameters in the participants is presented in Table S3. No significant relationship was detected between the 5000-m PBR and any of the physiological parameters (Table S3).

### 3.2. Comparison of Physiological Parameters at Maximal Exercise Between the AA Genotype and the T-Allele Carriers

The physiological parameters observed during maximal exercise for the AA genotype and the T-allele carriers in the participants are presented in Table 2. None of the measured parameters differed significantly between the two genotypes.

### 3.3. Comparison of Physiological Parameters at Submaximal Exercise Between the AA Genotype and the T-Allele Carriers

**Figure 2:**
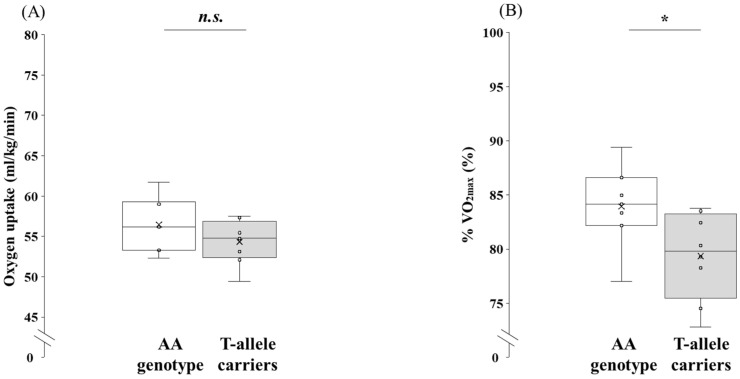
Comparison of $\overset{\cdot }{V}$O_2_-LT between the AA genotype and the T-allele carriers in the participants. $\overset{\cdot }{V}$O_2_-LT and %$\overset{\cdot }{V}$O_2max_ at LT were analyzed using an independent *t*-test. Box-and-whisker plots are presented with the median represented by a line, the mean by a cross, the box extending from the first to third quartiles, and whiskers indicating ±1.5 × the interquartile range. Individual data points are overlaid as circles. (**A**) $\overset{\cdot }{V}$O_2_-LT, oxygen uptake at the lactate threshold (relative value), and (**B**) %$\overset{\cdot }{V}$O_2max_ at LT, percentage of $\overset{\cdot }{V}$O_2_-LT relative to $\overset{\cdot }{V}$O_2max_.; AA genotype: white box whisker plot; T-allele carriers (AT + TT genotypes): gray box whisker plot. *: *p* < 0.05; n.s., not significant.

**Figure 3:**
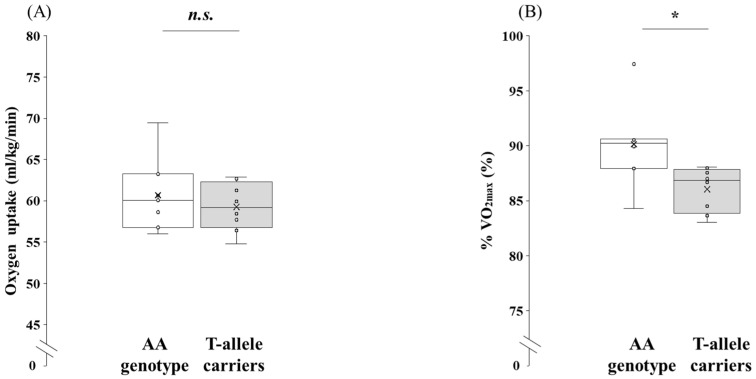
Comparison of $\overset{\cdot }{V}$O_2_-OBLA between the AA genotype and the T-allele carriers in the participants. $\overset{\cdot }{V}$O_2_-OBLA and %$\overset{\cdot }{V}$O_2max_ at OBLA were analyzed using an independent *t*-test. Box-and-whisker plots are presented with the median represented by a line, the mean by a cross, the box extending from the first to third quartiles, and whiskers indicating ±1.5 × the interquartile range. Individual data points are overlaid as circles. (**A**) $\overset{\cdot }{V}$O_2_-OBLA, oxygen uptake at the onset of blood lactate accumulation (relative value), and (**B**) %$\overset{\cdot }{V}$O_2max_ at OBLA, percentage of $\overset{\cdot }{V}$O_2_-OBLA relative to $\overset{\cdot }{V}$O_2max_.; AA genotype: white box whisker plot; T-allele carriers (AT + TT genotypes): gray box whisker plot. *: *p* < 0.05; n.s., not significant.

Physiological parameters recorded during submaximal exercise in the participants for the AA genotype and the T-allele carriers are shown in Figure 2 and Figure 3. No significant difference was observed between the two genotypes for $\overset{\cdot }{V}$O_2_-LT (*p* = 0.208, 95% CI: −1.36 to 5.69, *d* = 0.69). By contrast, %$\overset{\cdot }{V}$O_2max_ at LT was significantly higher in the AA genotype than in the T-allele carriers (*p* = 0.044, 95% CI: 0.15 to 9.02, *d* = 1.16).

No significant difference was observed between the two genotypes for $\overset{\cdot }{V}$O_2_-OBLA (*p* = 0.478, 95% CI: −2.80 to 5.67, *d* = 0.38). By contrast, %$\overset{\cdot }{V}$O_2max_ at OBLA was significantly greater in the participants with the AA genotype than in the T-allele carriers (*p* = 0.023, 95% CI: 0.67 to 7.50, *d* = 1.34).

### 3.4. Comparison of SmO_2_ at Maximal Exercise Between the AA Genotype and the T-Allele Carriers

**Figure 4:**
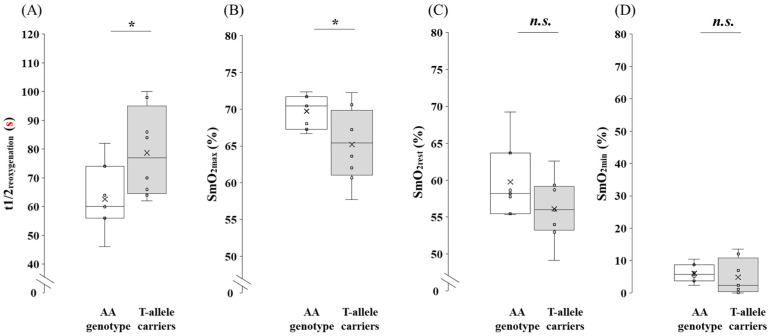
Comparison of SmO_2_ during maximal exercise between the AA genotype and the T-allele carriers in the participants. (**A**) t1/2_reoxygenation_, (**B**) SmO_2max_ and (**C**) SmO_2rest_ were compared using the independent *t*-test. (**D**) SmO_2min_ was analyzed using the Mann–Whitney U-test. Box-and-whisker plots are presented with the median represented by a line, the mean by a cross, the box extending from the first to third quartiles, and whiskers indicating ±1.5 × the interquartile range. Individual data points are overlaid as circles. AA genotype: white box whisker plot; T-allele carriers (AT + TT genotypes): gray box whisker plot. *: *p* < 0.05; n.s., not significant.

SmO_2_ values at maximal exercise for the AA genotype and the T-allele carriers in the participants are shown in Figure 4. The t1/2_reoxygenation_ was significantly shorter in participants with the AA genotype compared with the T-allele carriers (*p* = 0.043, 95% CI: −31.74 to −0.62, *d* = 1.16). SmO_2max_ was significantly greater in the AA genotype than in the T-allele carriers (*p* = 0.045, 95% CI: 0.13 to 8.96, *d* = 1.13). No significant differences were observed for SmO_2rest_ (*p* = 0.148, 95% CI: −1.47 to 8.78, *d* = 0.80) or SmO_2min_ (*p* = 0.336, 95% CI: −4.58 to 6.08, *r* = 0.27) between the genotypes.

### 3.5. Comparison of SmO_2_ at Submaximal Exercise Between the AA Genotype and the T-Allele Carriers

**Figure 5:**
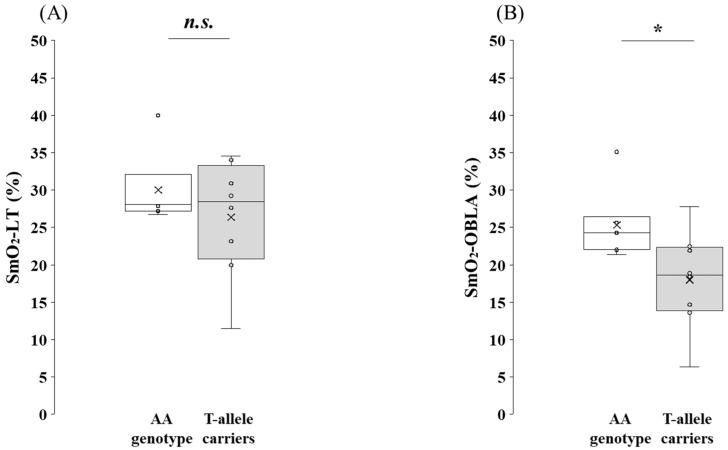
Comparison of SmO_2_ during submaximal exercise between the AA genotype and the T-allele carriers in the participants. (**A**) SmO_2_-LT and (**B**) SmO_2_-OBLA were analyzed using the Mann–Whitney U-test. Box-and-whisker plots are presented with the median represented by a line, the mean by a cross, the box extending from the first to third quartiles, and whiskers indicating ±1.5 × the interquartile range. Individual data points are overlaid as circles. SmO_2_-LT, muscle oxygen saturation at the lactate threshold; SmO_2_-OBLA, muscle oxygen saturation at the onset of blood lactate accumulation. AA genotype: white box whisker plot; T-allele carriers (AT + TT genotypes): gray box whisker plot. *: *p* < 0.05; n.s., not significant.

SmO_2_ values at submaximal exercise for the AA genotype and the T-allele carriers in the participants are shown in Figure 5. No significant difference was detected for SmO_2_-LT (*p* = 0.694, 95% CI: −3.75 to 10.73, *r* = 0.12). By contrast, SmO_2_-OBLA was significantly greater in the AA genotype carriers than in the T-allele carriers (*p* = 0.029, 95% CI: 0.57 to 13.20, *r* = 0.57).

## 4. Discussion

This pilot study aimed to compare physiological responses, including SmO_2_ during maximal and submaximal exercise, between the rs1049434 polymorphism genotypes in male collegiate LD runners. To our knowledge, this is the first study to examine the association between the rs1049434 polymorphism and SmO_2_ based on blood lactate values. The results showed that athletes with the AA genotype had shorter t1/2_reoxygenation_ and higher SmO_2max_ in the active muscle after maximal exercise than the T-allele carriers. In addition, those with the AA genotype exhibited significantly higher %$\overset{\cdot }{V}$O_2max_ at LT, %$\overset{\cdot }{V}$O_2max_ at OBLA, and SmO_2_-OBLA during submaximal exercise. These results suggest that LD runners with the AA genotype may have greater oxygen delivery capacity in active muscles, as reflected by relatively higher SmO_2_ at high exercise intensities and faster SmO_2_ recovery after maximal exercise.

Previous studies examining rs1049434 polymorphism in LD runners have been limited to two reports [19,20]. Ben-Zaken et al. demonstrated that the frequency of AA genotype was significantly higher among elite Ethiopian runners than among non-Ethiopian runners and controls [19]. Ben-Zaken et al. demonstrated that the prevalence of AA genotype was significant. Similarly, Seki et al. reported that Japanese male LD runners with 10,000-m PBRs under 28 min exhibited a significantly greater prevalence of AA genotype than those with slower PBRs [20]. Ben-Zaken et al. demonstrated that the frequency of AA genotype was significant. These findings suggest that rs1049434 polymorphism may represent a key genetic determinant of LD running performance. Accordingly, the present study aimed to elucidate the underlying physiological mechanisms by comparing skeletal muscle tissue-level responses to maximal and submaximal exercise between the rs1049434 genotype groups.

In this study, we found no significant differences between the rs1049434 genotypes in absolute measures, such as $\overset{\cdot }{V}$O_2max_, $\overset{\cdot }{V}$O_2_-LT, or $\overset{\cdot }{V}$O_2_-OBLA. However, the AA genotype exhibited significantly higher %$\overset{\cdot }{V}$O_2max_ at LT and %$\overset{\cdot }{V}$O_2max_ at OBLA than the T-allele carriers. Joáo et al. reported that Russian endurance athletes with AA genotype had a significantly higher $\overset{\cdot }{V}$O_2max_ than those with TT genotype [18]. Ben-Zaken et al. demonstrated that the frequency of AA genotype was significant [19]. Similarly, Seki et al. found that elite Japanese male LD runners with AA genotype showed significantly higher $\overset{\cdot }{V}$O_2max_, $\overset{\cdot }{V}$O_2_-LT, and $\overset{\cdot }{V}$O_2_-OBLA than T-allele carriers [20]. These findings differ from those of the present study. A likely explanation for the absence of genotype-related differences in $\overset{\cdot }{V}$O_2max_ in our cohort is the relatively homogeneous performance level of the participants. Legaz-Arrese et al. reported that differences in $\overset{\cdot }{V}$O_2max_ are typically not observed in athlete groups with small variations in performance [32]. Given the lack of a difference in $\overset{\cdot }{V}$O_2max_, the absolute values of $\overset{\cdot }{V}$O_2_-LT and $\overset{\cdot }{V}$O_2_-OBLA were also similar between genotypes in this study. By contrast, the higher %$\overset{\cdot }{V}$O_2max_ at LT and OBLA in individuals with the AA genotype may reflect enhanced oxygen utilization efficiency during exercise at moderate-to-high intensity. These results suggest that runners with the AA genotype tend to achieve a higher relative oxygen consumption at the same relative exercise intensity, supporting sustained aerobic metabolism. Furthermore, Gasser et al. reported that, in the general population, AA carriers exhibited a significantly higher proportion of type I muscle fibers than T-allele carriers [33]. This difference could contribute to relatively more efficient oxygen utilization in runners with the AA genotype.

Understanding the genetic influences of SmO_2_, a noninvasive marker of muscle oxygenation, is particularly relevant to LD runners. Physiological responses during exercise directly affect performance and provide a basis for training prescriptions and conditioning management. To date, only Flück et al. have examined the potential genetic effects of rs1049434 polymorphism on SmO_2_ [25]. In their study involving active military personnel and special operations soldiers, they reported a possible association between rs1049434 genotype and SmO_2_ at the first ventilatory threshold in the vastus lateralis and medial gastrocnemius, both before and during incremental exercise [25]. However, no prior studies have investigated SmO_2_ at lactate-referenced exercise intensities, such as at the LT or OBLA, which are widely recognized performance markers in LD runners. Additionally, no study has examined SmO_2_ in LD runners during exercises requiring high levels of oxygen delivery. In the present study, athletes with the AA genotype exhibited shorter t1/2_reoxygenation_ and higher SmO_2max_ following maximal exercise. These results indicate that runners with the AA genotype tend to achieve more rapid post-exercise reoxygenation in active muscles than the T-allele carriers. Clifford and Hellsten have reported that reoxygenation progresses when capillary perfusion is maintained, while mitochondrial oxygen consumption decreases after exercise [34]. Moreover, individuals with higher aerobic capacity generally demonstrate shorter t1/2_reoxygenation_ [29,35]. Considering that individuals with AA genotype have a higher proportion of type I muscle fibers, which are rich in mitochondria [33], these factors may facilitate rapid reoxygenation. By contrast, no differences were observed in SmO_2rest_ or SmO_2min_ between the genotypes. SmO_2rest_ values suggested that the oxygenation status of the vastus lateralis was comparable between AA genotype and T-allele carriers before the start of testing. Yogev et al. reported that SmO_2min_ during maximal exercise remained unchanged regardless of improvements in fitness following endurance training [36]. These findings indicate that changes in fitness level have little impact on SmO_2_ during maximal exercise, whereas changes during submaximal exercise may better reflect such physiological adaptations. Furthermore, the imbalance in local metabolism at higher intensities [22] may explain why SmO_2_ during maximal exercise remained unchanged before and after training. Taken together, these results suggest that runners with the AA genotype exhibit faster post-exercise reoxygenation and higher SmO_2_. These responses indicate that the genetic traits of the rs1049434 polymorphism are at least partly expressed during recovery.

Understanding muscle physiological responses during dynamic exercise is considered important for evaluating exercise intensity [37]. Furthermore, elucidating the limits of muscle oxygen utilization and the adaptations to training is fundamental to understanding exercise tolerance and functional capacity [38,39]. Real-time measurement of muscle oxygenation using NIRS has been recognized as a valuable tool for physiological assessment during training [40]. Blood lactate concentration is also considered a highly sensitive indicator of changes in exercise intensity and duration [41]. Importantly, a certain relationship between SmO_2_ measured by NIRS and blood lactate concentration has been reported, suggesting that exercise intensity assessment based on blood lactate can be improved by integrating SmO_2_ data. Regarding this relationship, Batterson et al. investigated the relationship between training indices referenced to blood lactate concentrations and SmO_2_ in 10 elite male soccer players [23]. They reported that the inflection points of blood lactate concentrations and SmO_2_ during an incremental exercise test were closely aligned [23]. In a related investigation, Athanasios et al. investigated the interrelationships among muscle oxygen saturation (SmO_2_), heart rate, and blood lactate (BLa) during interval training in 12 national-level swimmers (9 males and 3 females) and reported that these physiological variables changed in a correlated manner [37]. Based on these findings, the present study investigated the relationship between SmO_2_ and blood lactate-based indices, which are widely used for training intensity prescriptions and performance assessments in LD runners. The results demonstrated that the AA genotype exhibited higher SmO_2_ at OBLA than the T-allele carriers, whereas no significant differences were noted between the genotypes for SmO_2_ at LT. MCT1 is present not only in the plasma membrane but also in the mitochondrial inner membrane [42,43], where it serves an essential function in the transport of energy substrates such as lactate and pyruvate. MCT1 facilitates lactate transport across membranes by exploiting concentration gradients, thereby contributing to intracellular pH homeostasis and efficient energy flux [9,10,44]. Higuchi et al. reported that the functional activity of MCT1 is enhanced when rs1049434 polymorphism substitutes glutamic acid with alanine, inducing conformational changes that promote lactate transport [45]. Based on these cellular mechanisms, runners with the AA genotype may sustain a smooth energy supply to muscle tissue even under high-intensity exercise, preserving the balance between oxygen delivery and utilization. This could confer advantages in sustaining workloads above the LT, delaying fatigue, and improving performance in events requiring prolonged high-intensity efforts. In contrast, no genotypic differences were observed at the LT. These results (SmO_2_-LT and OBLA) suggest that the rs1049434 polymorphism may play a functional role in lactate transport under high-intensity exercise conditions, where lactate accumulation increases. Therefore, at LT, where blood lactate accumulation is minimal, no differences between genotypes were observed. Although the sample size was small, these preliminary results indicate that the AA genotype may exhibit greater oxygen utilization efficiency and potentially enhanced skeletal muscle oxygen delivery during high-intensity exercise. These findings should be interpreted with caution, and further studies with larger cohorts are needed to confirm these observations.

This pilot study has a few limitations. First, the participants were limited to collegiate LD runners, which prevents the generalization of the findings to elite runners. Bıçakçı et al. reported that using a homogeneous cohort can reduce variability in performance-related measures when examining genetic influences [46]. However, the inclusion of diverse populations is necessary to enhance generalizability [45]. Second, the sample size was small. Although we observed differences in SmO_2_ between genotypes, larger cohorts are needed to confirm these findings. Furthermore, the post hoc power analyses indicated that the achieved statistical power was below the conventional criterion of 0.80, suggesting that caution is warranted when interpreting the results. Third, this study employed a cross-sectional design with measurements taken at a single time point. Future research should incorporate longitudinal or training intervention studies to investigate genotype-specific adaptations to exercise. Addressing these limitations could help clarify the practical significance of rs1049434 polymorphism and inform individualized training strategies for performance optimization.

## 5. Conclusions

This pilot study evaluated physiological responses, including SmO_2_, during maximal and submaximal exercise between genotypes of the rs1049434 polymorphism in male collegiate LD runners. The results showed the AA genotype tended to exhibit higher %$\overset{\cdot }{V}$O_2max_ at LT and OBLA than T-allele carriers. Furthermore, those with the AA genotype appeared to show shorter t1/2_reoxygenation_ after maximal exercise, as well as higher SmO_2max_ and SmO_2_-OBLA, than T-allele carriers.

These findings suggest that LD runners carrying the AA genotype of the rs1049434 polymorphism may have greater oxygen utilization efficiency and potentially enhanced skeletal muscle oxygen delivery during high-intensity exercise. However, these are preliminary results, and further studies with larger and more diverse cohorts are needed to confirm these observations.

## Figures and Tables

**Table 1:** Physical characteristics of participants and 5000-m PBRs.

	**All Participants (*n* = 15)**			**AA Genotype (*n* = 7)**			**T-Allele Carriers (*n* = 8)**		
	Mean	±	SD	Mean	±	SD	Mean	±	SD
Age (years)	20.6	±	0.9	20.6	±	0.8	20.6	±	1.2
Career (years)	6.9	±	1.9	7.3	±	1.6	6.5	±	2.4
Height (cm)	172.8	±	6.1	174.9	±	6.2	170.9	±	5.8
Weight (kg)	58.3	±	3.4	58.6	±	3.6	58	±	3.5
FM (kg)	5.5	±	1.8	5.4	±	2.2	5.5	±	1.6
LBM (kg)	52.8	±	3.2	53.2	±	4.2	52.5	±	2.3
%BF (%)	9.1	±	2.4	8.8	±	2.9	9.4	±	2.1
BMI (kg/m^2^)	19.6	±	1.1	19.2	±	1.0	19.9	±	1.2
Skinfold thickness of the vastus lateralis (mm)	6.4	±	1.2	6.3	±	1.1	6.5	±	1.3
5000-m PBRs (s)	889.6	±	22.0	883.1	±	18.8	895.3	±	24.3

Data are presented as mean ± standard deviation (SD). T-allele carriers, AT + TT genotype; FM, fat mass; LBM, lean body mass; %BF, body fat percentage; BMI, body mass index; PBRs, personal best records.

**Table 2:** Comparison of physiological parameters during maximal exercise between the AA genotype and the T-allele carriers in the participants.

Variables (Unit)	Genotypes (*n*)						*p*-Value	95% CI			*Effect Size*	95% CI		
	AA Genotype (*n* = 7)			T-Allele Carriers (*n* = 8)										
$\overset{\cdot }{V}$O_2max_ (mL/kg/min)	67.3	±	3.3	68.6	±	4.4	0.543	−5.64	to	3.11	*d* = 0.32	−1.34	to	0.71
$\overset{\cdot }{V}$CO_2max_ (mL/kg/min)	76.3	±	5.5	81.4	±	5.8	0.109	−11.37	to	1.30	*d* = 0.89	−1.94	to	0.20
HR_max_ (beats/min)	191.0	±	9.1	188.8	±	6.0	0.578	−6.27	to	10.77	*d* = 0.30	−0.73	to	1.31
Respiratory Quotient	1.3	±	0.1	1.2	±	0.1	0.512	−0.07	to	0.13	*d* = 0.35	−0.68	to	1.37
VE_max_ (mL/kg/min)	2.9 (2.5 to 3.5)			2.7 (2.3 to 4.4)			0.536	−0.26	to	0.54	*r* = 0.18			
RPE	19.0 (18.0 to 20.0)			19.5 (17.0 to 20.0)			0.779	−2.00	to	1.00	*r* = 0.08			
BLa_max_ (mmol/L)	11.4	±	1.1	11.6	±	1.4	0.711	−1.69	to	1.18	*d* = 0.20	−1.21	to	0.83

$\overset{\cdot }{V}$O_2max_, $\overset{\cdot }{V}$CO_2max_, HR_max_, respiratory quotient, and BLa_max_ were analyzed using the independent *t*-test, and values are presented as means ± standard deviation. VE_max_ and RPE were analyzed using the Mann–Whitney U-test and presented as median (minimum to maximum). Effect sizes are reported as Cohen’s *d* (parametric) or *r* (non-parametric). T-allele carriers, AT + TT genotype; $\overset{\cdot }{V}$O_2max_, maximal oxygen uptake (relative value); $\overset{\cdot }{V}$CO_2max_, maximal carbon dioxide output (relative value); HR_max_, maximal heart rate; VE_max_, maximal ventilation (relative value); RPE, rate of perceived exertion; BLa_max_, maximal blood lactate concentration; CI, confidence interval.

## Data Availability

The data presented in this study are available from the corresponding author due to their sensitive nature. As the dataset contains privacy-sensitive details, it cannot be made publicly accessible in an open-access format.

## Supplementary Materials

The following supporting information can be downloaded at: https://www.mdpi.com/article/10.3390/genes16111324/s1, Figure S1: Placement of the MOXY sensor on the right vastus lateralis; Table S1: Normality test results for each physiological parameter according to the rs1049434 polymorphism of the *SLC16A1 (MCT1)* gene; Table S2: Post-hoc power analysis for physiological parameters according to the rs1049434 polymorphism of the *SLC16A1 (MCT1)* gene; Table S3: Correlations between the 5000-m PBRs and physiological parameters in long-distance runners.
