# Supplementary figures and images for: Muscle Oxygen Saturation Responses During Maximal and Submaximal Exercise According to SLC16A1 (MCT1) Gene Polymorphism in Long-Distance Runners: A Cross-Sectional Pilot Study
Source: Genes (Basel). 2025 Nov 3;16(11):1324. doi: 10.3390/genes16111324 (PMC12653002; doi:10.3390/genes16111324)

## Slide 1
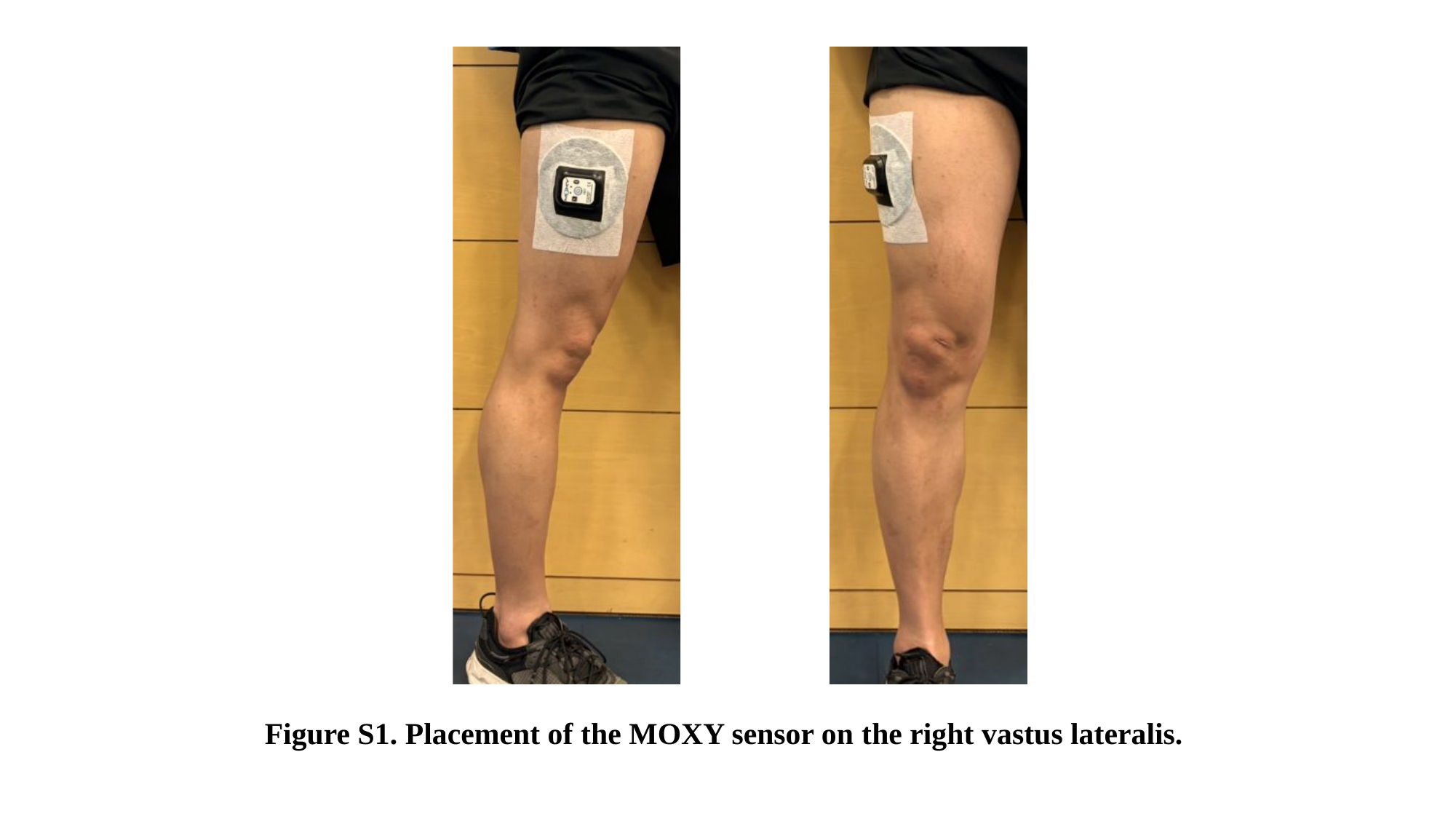

Figure S1. Placement of the MOXY sensor on the right vastus lateralis.

Supplement: Supplementary file 1 [file genes-16-01324-s001.zip › supplementary file.pptx]
